# Reduction of NrF2 as coadjuvant during the development of persistent periapical lesions

**DOI:** 10.4317/medoral.25815

**Published:** 2023-06-18

**Authors:** Carlos Guerrero-Bobadilla, Irinea Yáñez-Sánchez, Talia Franco-Ávila, Abril B Martínez-Rizo, Alfredo Domínguez-Rosales, Bertha Adriana Alvarez-Rodríguez, María Eugenia Vázquez-Sánchez, Rosa Arias-Gómez, Francisco Javier Gálvez-Gastélum

**Affiliations:** 1 Dental Clinics, CUCS, University of Guadalajara, Jalisco, Mexico; 2 Nanosciences and Nanotechnology Research Center, Department of Natural and Exact Sciences, CUValles, University of Guadalajara, Jalisco, Mexico; 3 Biomedical Research Laboratory, Academic Unit of Medicine, Autonomous University of Nayarit, Nayarit, Mexico; 4 Laboratory of Chronic Degenerative Diseases, CUCS, University of Guadalajara, Jalisco, Mexico; 5 Pathology Laboratory, Department of Microbiology and Pathology, CUCS, University of Guadalajara, Jalisco, Mexico

## Abstract

**Background:**

Persistent periapical lesions (PPL) are the result of pulpar necrosis induced by bacterial infection resulting in bone degradation and culminating with the loss of dental piece. Pathological changes in the peripapice are associated with the presence of free radicals. The transcription factor Nrf2 is the main regulator of the endogenous antioxidant response against oxidative stress and has been implicated in the regulation of osteoclastogenesis.The aim is to determine the oxidative condition in samples from patients with Persistent Periapical Injuries as a detonating factor of tissue damage.

**Material and Methods:**

An observational, descriptive, cross-sectional study was carried out in samples with PPL (cases) and samples by removal of third molars (controls) obtained in the clinic of the specialty in endodontics, University of Guadalajara. Samples were submitted to histological staining with Hematoxylin-Eosin, lipoperoxide analysis, Superoxide Dismutase (SOD), Glutathione-Peroxidase (GPx) and Catalase (CAT) activities were determined by immunoenzymatic assays and NrF2 by Western Blot analysis.

**Results:**

Samples from PPL patients histologically showed an increased presence of lymphocytes, plasma cells, and eosinophils, as well as a decrease in extracellular matrix proteins and fibroblast cells. There was a rise in lipid peroxidation, GPx and SOD activities, but an important decline (36%) in Catalase activity was observed (*p*<0.005); finally, NrF2-protein was diminished at 10.41%. All comparisons were between cases vs controls.

**Conclusions:**

The alterations in antioxidants endogenous NrF2-controlled are related to osseous destruction in patients with PPL.

** Key words:**Periapical lesions, NrF2, oxidative stress, antioxidant, granuloma, cyst.

## Introduction

The persistence of periapical lesions after endodontic treatment is associated with intraradicular and extraradicular infections, foreign body reaction, cyst formation, and fibrous scar tissue healing (1). Persistent periapical lesions (PPL) develop as an inflammatory response to pulp tissue necrosis to prevent the dissemination of bacteria and their toxins infection send towards the surrounding bone. Although PPL is a radiographic finding, histologically they are classified as periapical granulomas, radicular cysts, periapical scar, and other lesions (2). Commonly, an important proportion of cases (90%) clinically and radiologically diagnosed belong to the group of periapical granulomas and radicular cysts (2). However, the PPL have been analyzed from extracted non-root-filled teeth and the histopathological analysis of periapical lesions obtained during apical surgery (3). PPL are densely innervated, comprising an inflammatory exudate with abundant macrophages, lymphocytes, polymorphonuclear leukocytes, and plasma cells (4,5).

Bacteria play a major role in the etiology of periapical lesion formation resulting in bone resorption which is an active process carried out by osteoclasts (6) Several molecules and proteins are involved during this pathological process, as receptor activator of nuclear factor kappa B (NF-κB) and osteoprotegerin (OPG) that play crucial roles in regulating the differentiation, activation, and survival of osteoclasts (7).

Oxidative stress (free radicals generation) is involved in the pathogenesis of a variety of inflammatory disorders like periapical lesions, in particular the reactive oxygen species (ROS) produced by phagocytic cells in response to infectious process represent an important host defense mechanism, nevertheless in excess results in tissue injury and bond destruction (8) by activation of matrix metalloproteinases (MMPs) and inflammatory mediators through damage to deoxyribonucleic acids, proteins, lipids, and membranes (9). However, antioxidants present an opposing free radical action by both non-enzymatic and enzymatic reactions. Primary antioxidants are superoxide dismutase (SOD), glutathione peroxidase (GPx) (10). SOD removes superoxide radicals by catalyzing the dismutation of molecular oxygen and hydrogen peroxide. GPx is to scavenge hydrogen peroxide. Peroxide activation increases the levels of interleukin 6 (IL-6) and MMPs. Also, reduced levels of regulatory cytokines (e.g., transforming growth factor-1 (TGF-1) and interleukin 10 (IL-10) have been associated with continued inflammation of the ligaments and supporting tooth structures (11). Catalase (CAT) is an enzyme directly involved in active oxygen scavenging. Cat breaks down H2O2 to yield oxygen and water (12).

The nuclear factor-erythroid 2-related factor 2 (NrF2) is a protein involved in the cellular antioxidant defense system. Under normal conditions, Nrf2 exists in the cell, combined with Kelch-like ECH-associated protein 1 (Keap1). In response to ROS, Nrf2 is phosphorylated promoting its translocation into the nucleus for the upregulation of the expression of antioxidants and detoxifying genes by binding to antioxidant response elements (AREs) in the promoter region of the encoding genes, which starts the expression of phase II detoxifying enzymes and antioxidant genes, including Heme oxygenase-1 (HO-1), catalase (CAT), superoxide dismutase (SOD), gamma-glutamylcysteine synthetase (γ-GCS) and NAD(P)H: quinone oxidoreductase 1 (NQO1) (13-15). On one hand, NrF2/ARE activation may inhibit pro-inflammation, including cytokines, inflammatory chemokines, cell adhesion factors, and MMPs (15).

This paper aimed to determine the oxidative/antioxidant conditions in samples from patients with Persistent Periapical Injuries as a detonating factor of tissue damage and analyze the presence of NrF2 during the persistence of this pathology.

## Material and Methods

- Subjects and Sample Collection

An observational, descriptive, cross-sectional study was realized in 50 biological samples of periapical tissue obtained of patients with PPL (*n*= 30) determined according to American Association Endodontics criteria and histopathological analysis (16) or samples by removal of third molars (*n*= 20) without PPL were analyzed. Data of medical history was recorded, and no systemic disease was observed, and antibiotics had not been taken during the previous 6 months in any patients selected. Written consent was obtained from all patients prior to the collection of samples. Biological samples were collected from the patients who underwent apical microsurgery in the clinic of the specialty in endodontics, University of Guadalajara. The research protocol was approved by the Ethics Committee of CUCS, University of Guadalajara, number CI-22011 in accordance with provisions of the Declaration of Helsinki. Indications for apical microsurgery were based on single-rooted anterior teeth with persistent periapical lesions radiographically determined with observable radiolucent areas (0.5-1.0 cm in diameter) around the apex.

Periapical tissues were obtained from the patients at the time of apicoectomy to remove periapical lesions. Immediately, the tissues were cut into 2 portions at the center. One portion was fixed with 4% paraformaldehyde in phosphate buffered saline for histopathological evaluation. Another portion was frozen at -80 C for molecular analysis.

- Histopathological analysis

All biological specimens were obtained during apical microsurgery. An operating microscope was used to provide the optimum magnification and illumination. The periapical lesions were removed with minimum tissue damage, and an excisional biopsy technique was used for evaluation of the specimens which were fixed in paraformaldehyde 4% and processed for paraffin embedding.

Specimens were sectioned serially in thickness 5m using a microtome. The sections were stained with hematoxylin-eosin using standard techniques performed by experienced laboratory technicians, and they were examined under a light microscope (Axio, Zeiss) by a pathologist according to the histopathological criteria. Periapical granulomas were determined when the lesions were predominantly infiltrated with lymphocytes, plasma cells, or macrophages, with or without epithelial remnants, and had a surrounding capsule of collagen fibers. When a mass of polymorphonuclear leucocytes dominated the granulomatous tissue as a collection of pus (dead and dying neutrophils), the lesion was classified as a periapical abscess. Lesions with dense collagenous connective tissue and a lack of inflammatory cells were established as scar tissue. If there was a layer of stratified squamous epithelium along a surface of conjunctive tissue to indicate a delineated cavity and surrounded by a slight fibrous capsule, the lesion was diagnosed as a periapical cyst.

- Oxidative Stress

The ROS presence was determined by levels of lipid peroxidation (LPO) using a commercially available kit (FR12; Oxford Biomedical Research, Oxford, MI, USA) according to the manufacturer’s instructions. This assay is based on the reaction of the chromogenic reagent N-methyl-2-phenylindole with malondialdehyde (MDA) and 4-hydroxyalkenals (4HDA). Briefly, 0.2 mL homogenate (1 gr of tissue) was added to 10 L BHT (0.5 M) to prevent sample oxidation and were centrifuged at 3,000 x g for 10 minutes (4C). In 200 L of sample homogenated add 600 L of N-methyl-2-phenylindol in acetonitrile (Reagent 1) mixed in 150 μL of HCl (12 N). Incubate at 45C for 60 minutes. After that, centrifuge the mix reaction at 15,000 x g for 10 minutes and read at 586 nm using analyzer equipment (ELx800, Biotek). Standard curves using known concentrations of the standard 1,1,3,3-tetramethoxypropane in Tris-HCl were constructed.

- NrF2 analysis

The protein content in cytoplasm and nucleus was determined according to Andrews (17) using 100 mg of dental tissue that was homogenized in 800 μL of Hepes pH 7.9, KCl 10 mM, EDTA 0.1 mM, EGTA 0.1 mM, MgCl2 1.5 mM, and 33 µL protease inhibitors (A solution) in manual equipment (Pellet Pestle Motor Kontes, EU). The homogenate was centrifuged at 1 4000 ×g for 5 minutes at 4° C in 50 μL of NP-40 (10%), briefly supernatant (cytoplasmic proteins) was cooled at -70 C. The pellet (nuclear proteins) was resuspended in 300 µL of B solution (Hepes 20 mM pH 7.9, NaCl 0.4 M, EDTA 1 mM, EGTA 1 mM, MgCl 1.5 mM y 13 µL protease inhibitors and centrifugated 14000 x g for 5 mins at 4° C, finally the supernatant was recollected (nuclear proteins) and was cooled at -70°C. Both proteinic solutions (cytoplasmic and nuclear) were quantified according to Bradford (18). In 15 µg of proteins using β-mercaptoethanol (reducing agent) at 95 C during 5 mins were deposited in Sodium dodecyl sulfate-polyacrylamide gel electrophoresis (SDS-PAGE) at 12.5%, running at 100 V at 4 C for 3 h and the proteins were immune-transferred to a polyvinylidene fluoride membrane -PVDF- (Bio-Rad Laboratories, CA) at 200 V overnight at 4 C. The PVDF membrane was washed twice in tris buffered saline (TBS) and blocked with 5% of non-fat milk used for 1 h at room temperature with constant stirring. The membrane was then incubated for 1 h at room temperature with primary antibodies against NrF2, 110 kDa, MAB3925, R&D Systems 1:300) or -Actin, 43kDa, (Santa Cruz Biotechnology, sc-47778, United States, 1:1000). Membranes were then incubated for 30 min with an HRP-conjugated anti-rabbit secondary antibody in each case (1:1000 NRF2 y 1:8000 Actin). The signal was detected with the chemiluminescence Western Blotting kit (Roche Diagnostic, IN, USA) according to the manufacturer’s instructions. Relative units absorbance (RUA) were calculated from densitometric values of NrF2 and β-actin using the software Image Studio Lite 5.2.

- GPx, SOD determination

Glutathione Peroxidase is a tetramer of four identical subunits, with a molecular weight of 84 kD. GPx helps to prevent lipid peroxidation of cell membranes by consuming free peroxide in the cell. GPx was determined according to Glutathione peroxidase activity kit (ADI-900-158, Enzo Life Science), briefly, a homogenate of dental tissue was realized minced 1 gr of tissue in 10 mL of 1X assay Buffer (0.4 mM PMSF and 1% Triton X-100), centrifugated at 10,000 x g for 10-20 minutes at 4°C. The supernatant was deposited in a fresh tube prechilled on ice. The determination of GPx was performed at 340 nm in a final volume of 200 L (140 L of buffer 1X serum bovine albumin, 20 L of mix reaction 10X glutathione reductase, 20 L of proteins and 20 L of oxidant agent (cumene hydroxide) for 15 minutes. The results are represented as Units by milliliter (U/mL).

The activity of SOD was realized according to the activity assay kit (ab65354, Abcam) using 10 mg of dental tissue perfused by 150 mM of KCL and homogenized with 0.1M Tris/HCl, pH 7.4 (0.5% Triton X-100, 5mM β-ME, 0.1 mg/ml PMSF) in ice-cold as centrifugated at 14,000 x g for 5 minutes at 4 C. The supernatant (total SOD activity) was transferred to a clean tube kept of ice. The analysis of samples (20 L) was accomplished at 450 nm on a microplate reader, the SOD activity is represented as U/mg.

- CAT analysis

Total proteins were determining according to Bradford (1976), briefly 150 mg of dental tissue was minced in 400 μl of protein extraction buffer, containing 0.05M Tris-HCl, NaCl2 0.15M, HEPES 0.01M, CaCl2mM, Tween 80 0.01%, PMSF- 1mM, pH 8.5). The homogenate was centrifuged at 12,000 × g for 15 minutes at 4°C in a micro-tube to remove particulate matter and the supernatant was 1:20 diluted in PBS and stored at −70° C. All samples were minced in 2 mL of Bradford (Bio-Rad) for 20 minutes, finally the absorbance was determined at 595 nm (18). Cat activity was analyzed using 150 μg of total protein which were electrophoresed on 9.5% SDS-PAGE and developed using a buffer containing a mixture of 15 ml 60 mM Na-thiosulfate, 35 mL 3% H (Sol. A), and 50 ml 90 mM potassium iodide and 250 ml of glacial acetic acid (Sol. B). Sol. A was quickly mixed before pouring it onto the gel; this was then incubated for 30 seconds. After that, Sol. A was drained, and Sol. B added. Following incubation, the gels were rinsed and placed in deionized water. This is a negative staining assay; hence, Cat activity results in achromatic spots on a dark background. Activity in the gel slabs was quantified (all tests of zymography were measured and represented in relative units area using an image analyzer system (Kodak 1D 3.5 image analyzer). Activities in the gel slabs were quantified as relative units absorbance (RUA).

- Statistical analysis

The results are expressed as means and standard deviations. To compare results, the Student’s t test was used for continuous variables. SPSS for IBM statistical software (version 20 for Windows; IBM Corp., Armonk, NY, USA) was used for data analysis.

## Results

- Histopathological analysis

**Figure 1:**
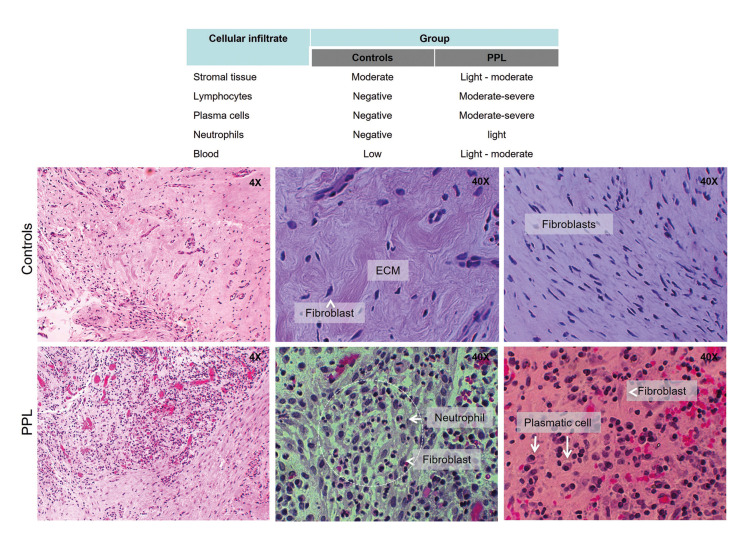
Histopathological analysis. Histological features in periapical lesions and cellular infiltrate on tissue of PPL patients.

Of the two groups analyzed, only in the patients with PPL a high activity of immune cells was observed, represented mostly by lymphocytes (moderate to severe), plasma cells (moderate to severe) followed by neutrophils to a lesser extent degree (slight) respect to patients that non presented the lesson, as shown in Fig. 1. Similarly, the PPL group show abundant inflammatory infiltrate, marked vascular congestion, and disorganization of extracellular matrix (stroma) compared with the control group was seen the alignment of fibroblast with tapered and elongated nuclei, free of inflammatory cells, dense collagen organized in a serpentine shape. Fibroblasts are very eosinophilic and difficult to distinguish their cytoplasm and cell-cell separation.

- Oxidative stress

**Figure 2:**
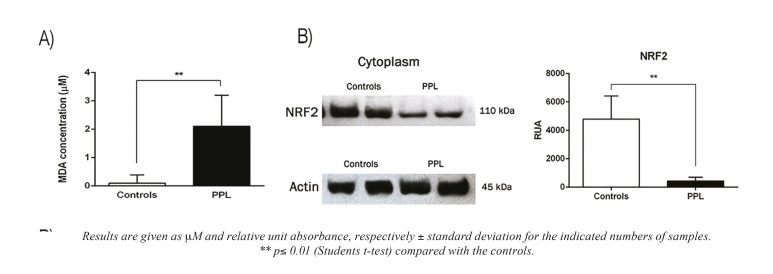
ROS and NrF2 determination. In A) Malondialdehyde levels and B) NrF2 presence.

Concerning oxidative stress analysis, our results indicated that the level of MDA was superior significantly in PPL than control patients (2.106 and 0.097 M, respectively) as shown in Fig. 2.

- NrF2 determination

To analyze the cytoprotective effect against oxidative stress, the presence of NrF2 was analyzed in cytoplasm with a significative decrement of NrF2 (89.59%) was observed in PPL-patients (p≤0.001). This result suggest that the NrF2-dependent effect cytoprotective enzyme expression may be attenuated in this same group respect to controls Fig. 2.

- Enzyme antioxidants analysis in controls and PPL samples

**Figure 3:**
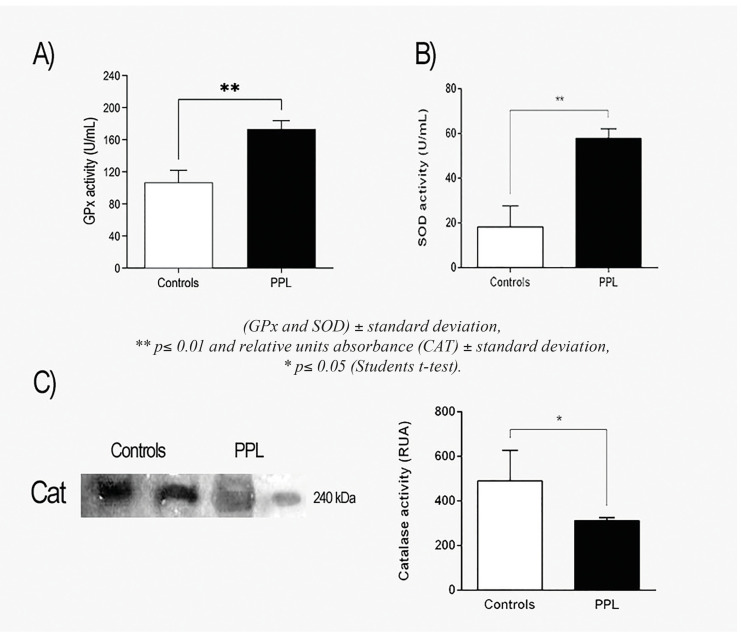
Antioxidants enzymes. A) GPx activity, B) SOD activity and C) Catalase activity. Results are given as U/mL

The level of GPx (102.9 U/mL) was higher (*p*<0.05) in periapical lesion samples than in control samples (167.8 U/mL) as show in Fig. 3. Similarly, the activity of SOD was increased in PPL than control samples (57.934 and 18.341 U/mL, respectively) see Fig. 3. On the side, an important decrement of the CAT activity was observed in dental tissue of patients with PPL with respect to controls (487 and 312 RUA, respectively) as observed in Fig. 3.

## Discussion

During our histopathological analysis, the presence in greater proportion by lymphocytes, plasma cells followed by neutrophils was observed in PPL samples. Similar results were reported by Neville *et al*., who indicated the presence of a chronic inflammatory reaction in the periradicular region that consist of a mass of granulation tissue surrounded by fibrous connective tissue, represent by lymphocytes associated with plasma cells, neutrophils, histiocytes, eventual mast cells, and eosinophils (19). Also, we observed the presence of abundant inflammatory infiltrate, vascular congestion, and disorganization of extracellular matrix (stroma) in the PPL group. Altaie *et al*., indicate an important presence of natural killer cells, dendritic cells, pro-inflammatory M1 macrophages, and anti-inflammatory M2 during a metabolomic analysis in periapical lesions and proposed different genes and their importance in the initiation and progression of each periapical lesion (periapical granuloma, periapical cysta and abseces) (20).

Oxidative stress is involved in the pathogenesis of a variety of inflammatory disorders. Periapical lesions usually result in the formation of an osteolytic lesion caused by the immune response to endodontic infection. ROS produced by phagocytic cells in response to bacterial challenge represents an important host defense mechanism, nonetheless disturbed redox balance results in tissue injury. Oxidants can cause tissue injury via damage to deoxyribonucleic acid (DNA) and proteins, peroxidative injury to lipid membranes, activation of proinflammatory cytokines, and proteases like MMPs. When polyunsaturated fatty acids are oxidized by ROS, malondialdehyde (MDA) is produced upon fatty acid decomposition; thus, measurement of MDA has been used as an indicator of LPO (21). Oxidative stress has an important role in the pathogenesis during PPL formation, for example in a radicular cyst (9). The levels of lipid peroxidation by MDA analysis have been reported in several investigations as Franco C. (2017), in an experimental study were reported a significant elevation in the levels of MDA in periapical lessons (22). Similarly, our results are contundents with this author and others as showed by Marton IJ, (1993) that indicate an elevation of MDA (805±365 nmol/g protein) in periapical granuloma samples with respect to controls (469 + 202 nmol/ g protein) (23) or Malik *et al*. (2020), that report an increment of levels of MDA in patients with radicular cyst as compared to control group (4.11 and 1.37 nmol/ml, respectively) (9). All these results indicate that reactive oxygen radicals cause biologically significant lipid peroxidation in PPL.

On the other hand, the antioxidant status exhibits the ability of antioxidants to scavenge free radicals or the inactivation of the regulation internal mechanisms of antioxidants like NrF2. Antioxidant mechanisms involve both, enzymatic and non-enzymatic reactions. Primary enzymes are SOD, CAT, and GPx. SOD catalyzes the dismutation reaction of superoxide radical anion (O-) to hydrogen peroxide, which is then catalyzed to innocuous O and HO by GPx and CAT. Our results were contradictory in this aspect, to reported by Malik *et al*. (2020) since the levels of SOD and GPx (57.934 and 102.9 U/mL, respectively) were superior in PPL patients and they reported a significant decrement in patients compared to controls in levels of SOD (0.03 and 0.11 nmol/ml, respectively) and GPx (5.88 and 7.87 nmol/ml, respectively) (9) Probably by MDA may reduce the activity of glutathione peroxidase in periapical granulomas, as it has been reported by Ayala *et al*. (10). The reduced activity of SOD and GPx may further the development of the lesion, resulting in oxidative stress (9). However, it should be noted that these results were carried out in serum and ours in dental tissue samples. Respect to the role of CAT in maintaining periodontal health was previously demonstrated in acatalasemia patients who are systemically healthy but have an increased incidence of chronic periodontitis. In our analysis the CAT activity was significantly reduced in PPL samples to respect controls, this decrement in CAT activity was reported by Sima C, *et al*. but in oral neutrophils isolated of severe chronic periodontitis (24) in opposition to reported by Esposito P. *et al* that showed a significative increment of CAT activity in patients with inflamed pulp tissue specimens (25).

Finally, for NrF2 presence that regulates gene transcription of antioxidant and phase 2 detoxifying enzymes and plays a role against oxidative insults inducing cytoplasmic Nrf2 its released from Keap 1-like and binds to antioxidant response elements in the promoter region of many antioxidant enzymes as catalase (CAT), superoxide dismutase (SOD) heme-oxygenase 1 and glutathione peroxidase (GPx) and others (24,26). The current study demonstrates that the Nrf2 pathway is down-regulated in the tissue of patients with PPL compared with tissue in healthy controls. This NrF2 deficiency could are related to the induction of osteoclastic bone resorption (present in PPL patients) as has been reported in culture cells (27).

On the other hands, NrF2 has been involved in the transcription of several cytokines critical for mounting both inflammatory and tissue protective responses, as IL-6, IL-17 and IL-22 (28,29). Lin *et al*., report that Nrf2 promotes IL-22 production while it inhibits IL-17A expression in Th17 cells and suggest that an therapeutic strategy which inhibits inflammatory IL-17 (Th17 lymphocyte) responses but concomitantly promotes IL-22 dependent tissue protective or regenerative response is of great clinical significance. An important role of IL-17 on development of PPL has been reported by Colic M *et al*., indicating that IL- 17, by stimulating the production of IL-8, may play a role in exacerbating inflammation within chronic periapical lesions (30). Similar results show Andrade A, *et al*., and Altaie *et al*. demonstrates that Th17 cells interact at the site of injury, suggesting the involvement of pro-inflammatory (IL-17) cytokines in the pathogenesis of chronic periapical lesions by enhance bone resorption and stimulating osteoclasts (20). The mechanism by which NrF2 participates in the expression of afore mentioned cytokines is still unknown.

In our knowledge, the present study is the first to report an association between NrF2 participation in patients with PPL and opens the question of whether the respiratory brush, the presence of the immune cells (neutrophils mainly), IL-17 or if the difference in antioxidants effects may also be a reflect of differences between PPL patients and healthy controls in our study. Based on the above we concluded that antioxidant mechanisms pay a role during the development of PPL since they are related to immunological and microbial processes that culminate in bone destruction and dental organ loss.

According to clinical impact of our results, the decrease in the NrF2 factor could have an important participation in the regulation of oxidative stress and the contribution to the persistence of periapical lesions (as clinicopathologic factor). Therefore, more studies are required to determine biomarkers in the prognosis of these lesions. NrF2, can help to investigate new personalized therapeutic strategies to control chronic periapical lesions and thus improve the prognosis, determine the prevalence of this lesions by the polymorphism presence in the population or could indicate the pharmacological treatments or bioactive components in the diet thah contribute to balance redox NrF2-controled. NrF2 is a factor that could be used as a therapeutic target for ameliorates this oral pathology but are necessary several studies.
